# Supplementary material for: Investigation of Deoxidation Process of MoO3 Using Environmental TEM
Source: Materials (Basel). 2021 Dec 22;15(1):56. doi: 10.3390/ma15010056 (PMC8746121; doi:10.3390/ma15010056)
Supplement: Supplementary file 1 [file materials-15-00056-s001.zip › materials-1453832-supplementary.pdf]

Supplementary Materials

Investigation of deoxidation process of MoO_3 using environmental TEM

Peijie Ma[1], Ang Li[1,*], Lihua Wang[1] and Kun Zheng[1,*]

1 Beijing Key Lab of Microstructure and Properties of Solids, Faculty of Materials and Manufacturing, Beijing University of Technology, Beijing, 100124, China
* Correspondence: ang.li@bjut.edu.cn (A.L.); Tel.: 86-10- 67396349 (A.L.)

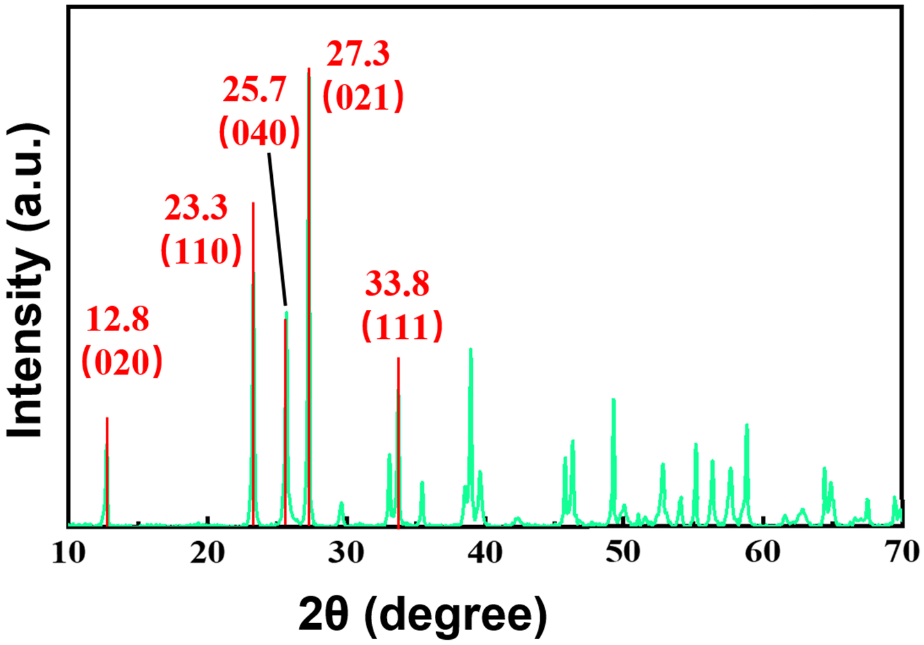

Figure S1. XRD patterns of MoO_3 nanosheets.

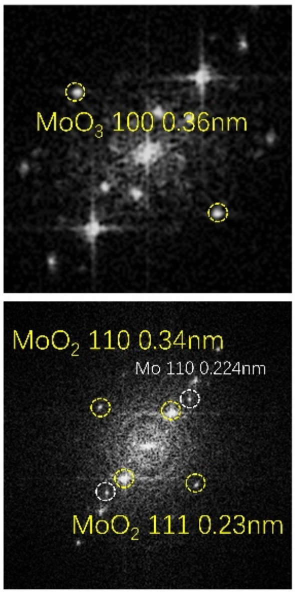

Figure S2. Diffraction calibration of the sample in Figure 3 at 800 °C.

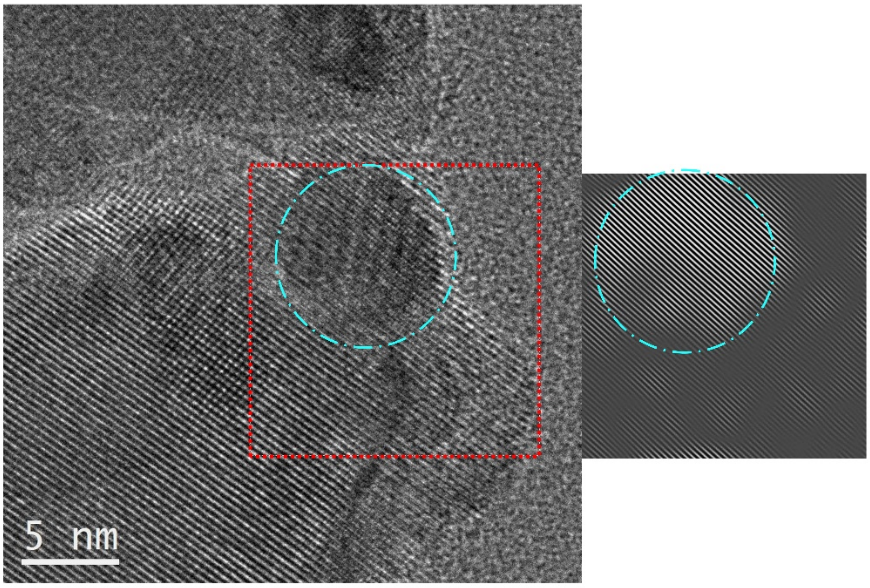

Figure S3. Filtering analysis of the sample in Figure 3 at 800 °C .
